# Supplementary material for: Identification of the 14-3-3 Gene Family in Bamboo and Characterization of Pe14-3-3b Reveals Its Potential Role in Promoting Growth
Source: Int J Mol Sci. 2022 Sep 23;23(19):11221. doi: 10.3390/ijms231911221 (PMC9569445; doi:10.3390/ijms231911221)
Supplement: Supplementary file 1 [file ijms-23-11221-s001.zip › Figure supplementary.pdf]

Supplementary Figures

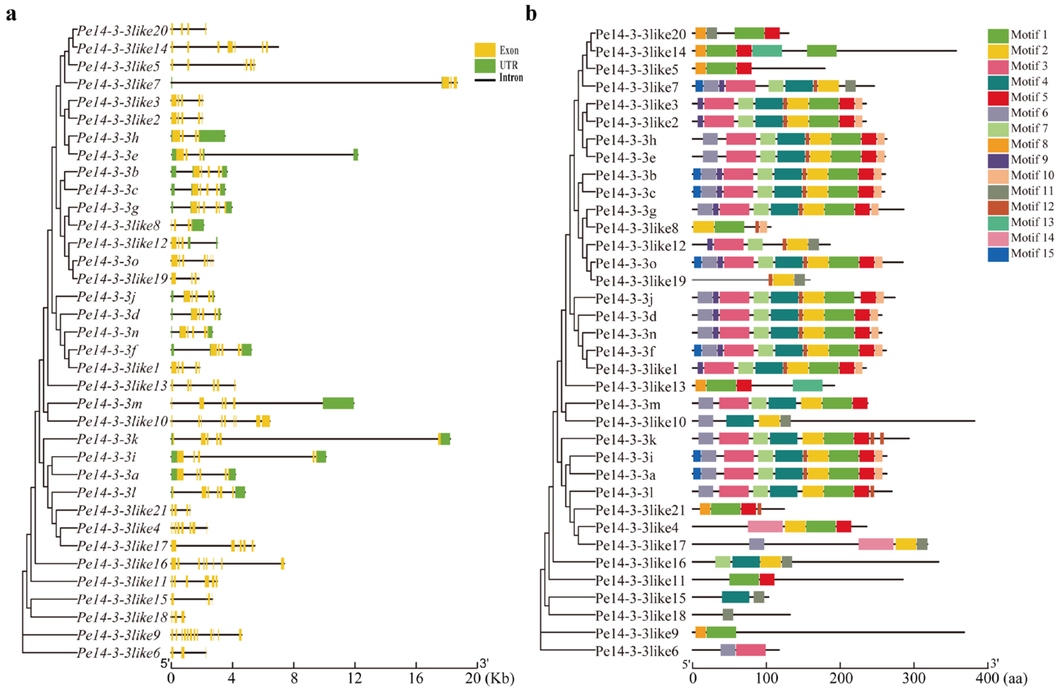

Figure S1. Structural analysis of *Pe14-3-3*s (**a**) Gene structures of *Pe14-3-3*s. The yellow squares, green squares, and black lines represent the exon, untranslated region (UTR), and intron, respectively. (**b**) Conserved motifs of Pe14-3-3s. Different motifs are represented in different color blocks.

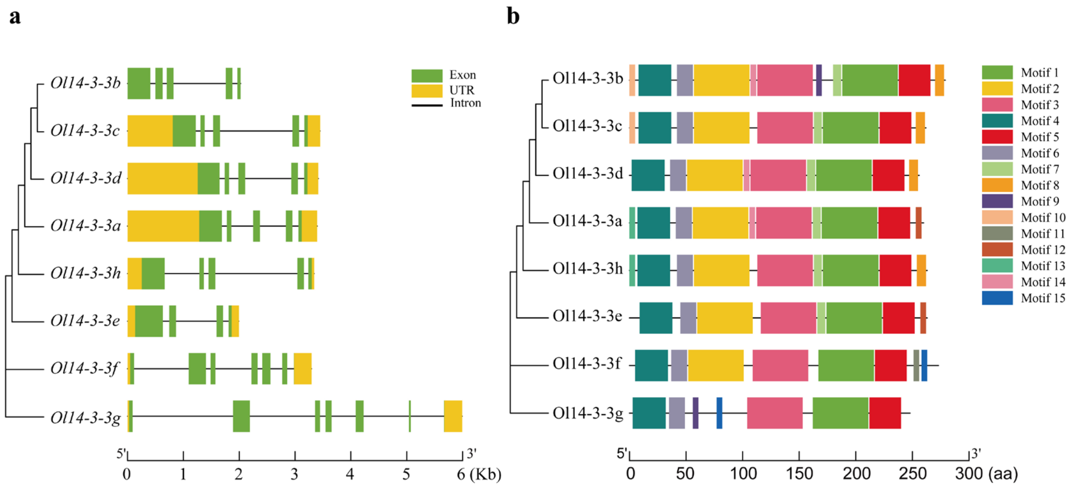

Figure S2. Structural analysis of *Ol14-3-3*s (**a**) Gene structures of *Ol14-3-3*s. The green squares, yellow squares, and black lines represent the exon, untranslated region (UTR) and intron, respectively. (**b**) Conserved motifs of Ol14-3-3s. Different motifs are represented in different color blocks.

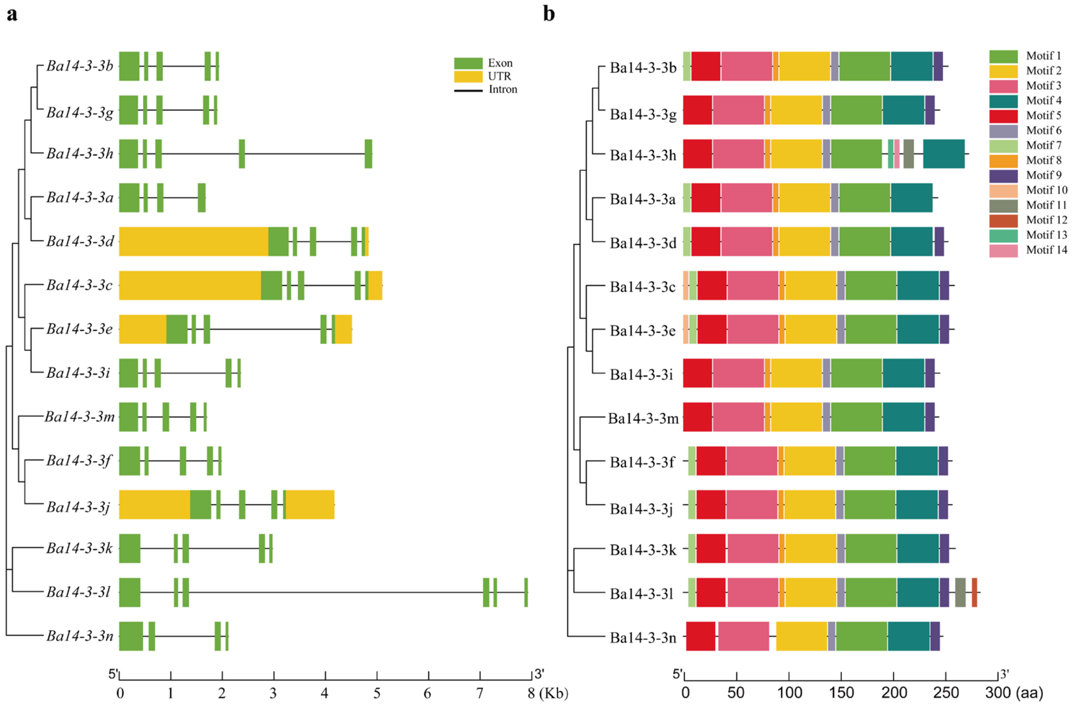

Figure S3. Structural analysis of *Ba14-3-3*s (**a**) Gene structures of *Ba14-3-3*s. The green squares, yellow squares, and black lines represent the exon, untranslated region (UTR), and intron, respectively. (**b**) Conserved motifs of Ba14-3-3s. Different motifs are represented in different color blocks.

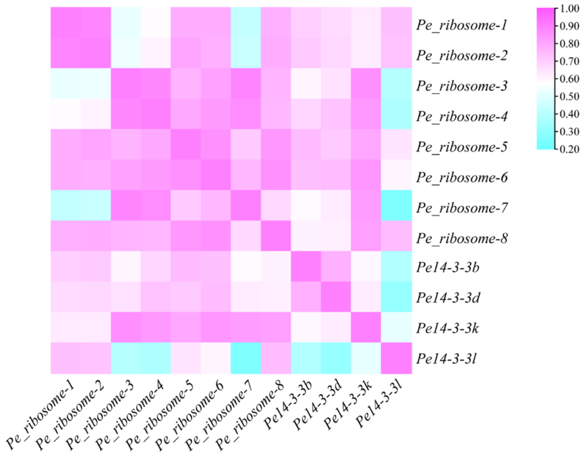

Figure S4. The correlation analysis of 12 genes (four *Pe14-3-3*s and eight *Pe_ribosome*s) based on the expression levels in the shoots with different heights and those in the roots with different lengths of *P. edulis*. Each correlation is shown by the colored squares from blue to purple which indicate the positive correlations from low to high.

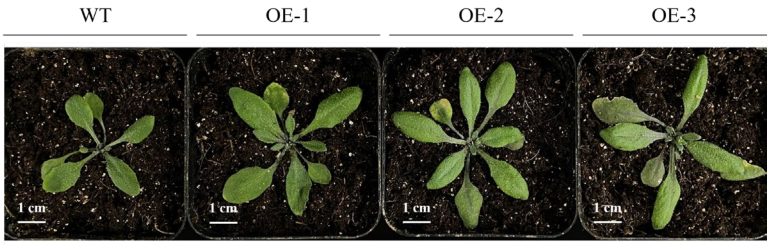

Figure S5. The leaf phenotypes of transgenic lines and WT plants. The photos were taken after germination for three weeks. Bars = 1 cm.
